# Clinical, Histological, and HPV-Related Factors Associated to Diffuse Presentation of Exophytic Nasal Papillomas

**DOI:** 10.3390/jcm13226638

**Published:** 2024-11-05

**Authors:** Marta Fulla, Beatriz Quiros, Omar Clavero, Montse Gomà, Álvaro de Andrés-Pablo, Miquel Àngel Pavon, Anna Penella, Laia Alemany, Xavier González-Compta, Marisa Mena

**Affiliations:** 1 Department of Otorhinolaryngology, Hospital Universitari Bellvitge, 08907 L’Hospitalet de Llobregat, Spain; apenella@bellvitgehospital.cat (A.P.); xgonzalez@bellvitgehospital.cat (X.G.-C.); 2 Program of Molecular Mechanisms and Experimental Therapy in Oncology, Bellvitge Biomedical Research Institute (IDIBELL), 08907 L’Hospitalet de Llobregat, Spain; mgoma@bellvitgehospital.cat; 3 Cancer Epidemiology Research Program, Catalan Institute of Oncology (ICO), 08907 L’Hospitalet de Llobregat, Spain; bquiros@iconcologia.net (B.Q.); oclavero_ext@iconcologia.net (O.C.); adeandres@idibell.cat (Á.d.A.-P.); mpavon@iconcologia.net (M.À.P.); lalemany@iconcologia.net (L.A.);; 4 Epidemiology, Public Health, Cancer Prevention and Palliative Care Program, Institut d’Investigació Biomèdica de Bellvitge (IDIBELL), 08907 L’Hospitalet de Llobregat, Spain; 5 Centro de Investigación Biomédica en Red de Cáncer (CIBERONC), Instituto de Salud Carlos III, 28029 Madrid, Spain; 6 Department of Pathology, Hospital Universitari Bellvitge, 08907 L’Hospitalet de Llobregat, Spain; 7 Centro de Investigación Biomédica en Red de Epidemiología y Salud Pública (CIBERESP), Instituto de Salud Carlos III, 28029 Madrid, Spain; 8 Faculty of Medicine, University of Barcelona, 08907 Barcelona, Spain

**Keywords:** rhinology, surgery of the paranasal sinuses, clinical research, nose and paranasal sinuses, cohort study

## Abstract

**Background**: Sinonasal exophytic papillomas (SNEP) are benign tumours arising from nasal mucosa. Human papillomavirus (HPV) infection seems to be related to the aetiology of a fraction of SNEP cases. SNEP presentation can be focal (FSNEP) or diffuse (DSNEP), but factors related to focal or diffuse presentation have not yet been well ascertained. This study aimed to analyse clinical, histological, and HPV-related differences between FSNEP and DSNEP. **Methods**: A retrospective cohort of 18 patients with SNEP from our centre were evaluated. Demographic, clinical and follow-up data were collected. All samples were subject to histopathological evaluation, DNA quality control, HPV-DNA detection, and viral load assessment. Univariate analyses were performed to evaluate differences between FSNEP and DSNEP. **Results**: Twelve SNEP patients were included in the final analysis. Seven patients had a diffuse nasal presentation, being younger than patients affected with FSNEP (42.7 years vs. 65.2 years, *p* = 0.019). The nasal septum was significantly more affected in DSNEP than in FSNEP (85.7% vs. 20%, *p* = 0.029). HPV-DNA was detected more frequently (100%) in DSNEP (HPV11 in six cases, HPV6 in one case) than in FSNEP (40%, *p* = 0.045, HPV6 in two cases). The median viral load among HPV6-positive samples was 626.8 virus/cell for FSNEP and 80.2 for DSNEP, and among HPV11-positive samples was 1673.7 for DSNEP. Recurrences were more frequent in the diffuse than in the focal group (85.7% vs. 20%, *p* = 0.029). **Conclusions**: The diffuse presentation of SNEP seems to be related to younger patients, nasal septum involvement, HPV infection, mostly HPV11, and a higher risk of recurrence.

## 1. Introduction

Sinonasal papillomas, a group of benign epithelial neoplasms located at the sinonasal tract, are classified as exophytic (SNEP), inverted, and oncocytic papillomas according to their histological characteristics (WHO classification of head and neck tumours 4th Edition 2017) [1]. All types may coexist [2].

SNEPs, also known as everted, fungiform, septal, or transitional papilloma, are the second most diagnosed subtype after sinonasal inverted papilloma. They represent 20–45% of all sinonasal papillomas with an incidence of 0.17 per 100.000 inhabitants per year [3,4,5]. They are more frequently diagnosed in males and in younger populations [6]. 

SNEPs usually manifest as progressive unilateral nasal obstructions, but in some cases, they can affect bilaterally or multifocally and may cause a complete nasal blockage. Less frequently, they can produce epistaxis or be diagnosed as a lesion emerging through the nostril. They mostly affect the anterior part of the nasal septum, but can also involve the lateral wall and the paranasal sinus [6,7]. They can be found as a localised or focal SNEP (FSNEP) or as a multifocal nasal pathology, although the latter is poorly described in the literature [8,9,10]. The diffuse or multifocal SNEP (DSNEP) has a different behaviour due to the wide involvement of the nasal mucosa and bilateral affectation. DSNEP often require multiple surgeries, given the high recurrence rate, and a closer follow-up [8]. The factors responsible for the diffuse presentation are not well ascertained due to the relative low prevalence of DSNEP. Recurrence rates in SNEP are around 22% [3,6], and previous publications observed recurrence in 83.3% of diffuse and bifocal SNEPs versus no recurrences in FSNEPs [8]. Malignant transformation is exceptional [11,12], with a single reported series of five SNEPs including two cases progressing to squamous cell carcinoma [13].

**Figure 1:**
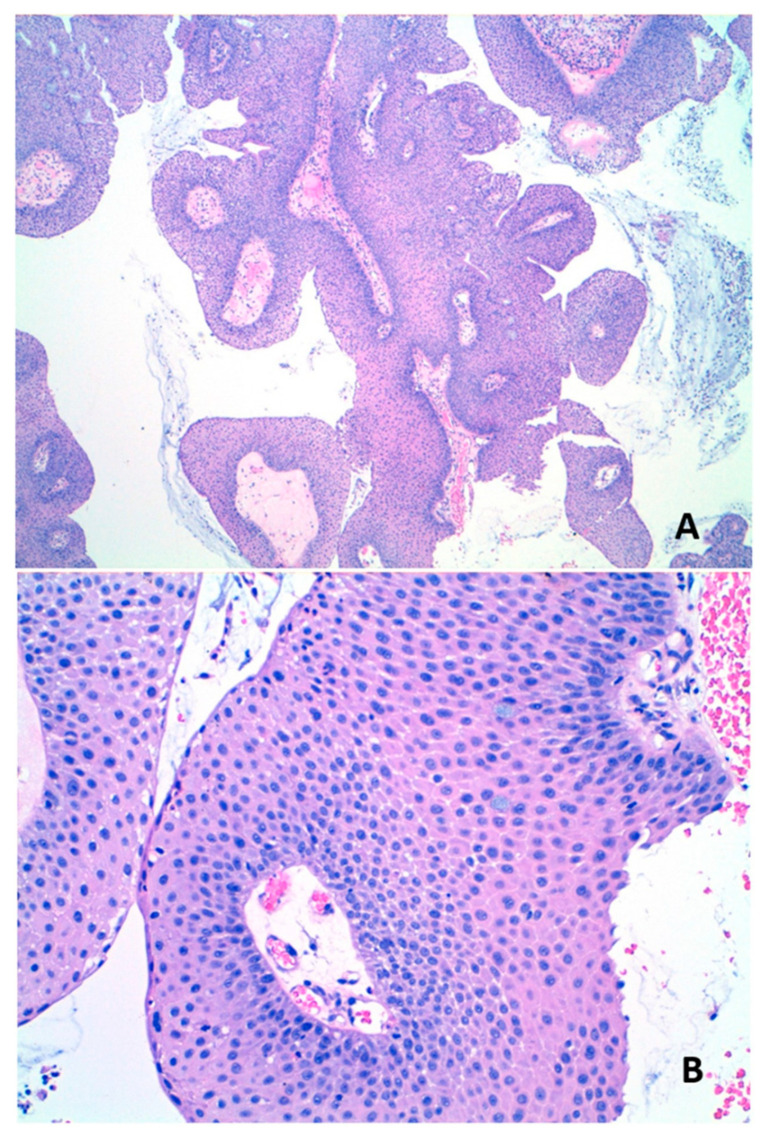
Sinonasal exophytic papilloma. (**A**) Squamous epithelium with exophytic growth pattern (hematoxylin-eosin 4×). (**B**) High power image shows non-keratinizing squamous epithelium and the presence of goblet cells with cytoplasmatic mucus (hematoxylin-eosin 20×).

Histologically, an SNEP is composed of branching papillary structures covered by non-keratinizing squamous or transitional epithelium. Interspersed mucin-secreting cells and intraepithelial mucous cysts are often identified (Figure 1). The main differential diagnosis is the inverted sinonasal papilloma that shows a typically endophytic or inverted growth pattern and the cutaneous squamous papilloma of the nasal vestibular skin. Unlike SNEPs, a skin squamous papilloma entirely comprises squamous cells and lacks intraepithelial mucocytes [2,7,14].

SNEP treatment is endoscopic surgical resection. However, in the diffuse form with multiple recurrences, the use of adjuvant drugs may decrease the percentage of relapse. DSNEP has similarities to recurrent respiratory papillomatosis (RRP): both are benign lesions related to HPV in the respiratory tract tending to present multiple recurrences. The local injection of cidofovir in DSNEP has been published recently with promising outcomes, despite small sample size [15]. The rationale for the use of bevacizumab in DSNEP is based in the recent RRP-related literature [16,17]. Chen et al. concluded that a submucosal nasal injection of bevacizumab is a safe treatment in hereditary hemorrhagic telangiectasia patients [18].

SNEP aetiology has not been fully elucidated. Lately, different agents have been reported as implicated in the development of sinonasal papillomas such as chronic sinonasal inflammation, drug use, or smoking. In SNEP, no association with chronic rhinosinusitis has been reported [3,8]. In the last decades, several studies have explored the relationship between sinonasal papillomas and human papillomavirus (HPV), mostly in inverted subtype, without confirming an etiological implication [19,20,21,22]. Detection rates of HPV in SNEPs are estimated to be around 60%, despite the heterogeneity of the data in the world literature, with a predominance of low-risk HPV genotypes (HPV 6 and 11) [3,5,8,23,24,25].

To the best of our knowledge, there has not yet been evidence on the relationship between the presence of HPV infection and focal or diffuse SNEP clinical presentation. 

The hypothesis of this study was that some clinical, histological, and HPV-related factors such as HPV-DNA prevalence, type distribution, and viral load could be related to the diffuse presentation of SNEP. 

## 2. Materials and Methods

### 2.1. Study Design

We conducted a retrospective study of a cohort of primary SNEPs consecutively diagnosed between 2013 and 2019 in our centre according to pathology department databases. Demographics and information about history of smoking, history of known HPV infection, immunosuppression, symptoms, localization in the nasal cavity, presentation (focal or diffuse), CT-scan findings, treatment, and follow-up were collected from medical records of otorhinolaryngology and pathology departments. We defined DSNEP as a multifocal lesion seen by nasal endoscopy and confirmed by biopsy. Unique lesions were classified as FSNEP. All cases were treated by endoscopic surgery using microdebrider and also, in DSNEP patients, with a local injection of bevacizumab. A complete resection without considering margins was performed in all cases.

Protocols were approved by our hospital ethics committee with the number PR413/20 and approval code ICO-VPH-PE-2020, which required informed consent to use archived samples, that were signed by the patients during the medical management and follow-up.

### 2.2. Formalin-Fixed, Paraffin-Embedded (FFPE) Blocks Processing and Histopathological Evaluation

FFPE blocks were processed under strict conditions to avoid contamination as previously described [26]. Briefly, four paraffin sections were selected for each FFPE block. Sections one and four were intended for histopathological evaluation after hematoxylin and eosin (H&E) staining and sections two and three for HPV testing and genotyping (sandwich method). Paraffin blocks were analysed under strict pre/post polymerase chain reaction (PCR) physical separation. To assess the DNA quality control, blank FFPE blocks were systematically processed in parallel as sentinels for contamination. Two pathologists designed a form specifically for the study (see Supplementary Material) for pathology review. The pathologists had no access to the original local diagnosis. After the first evaluation, a pre-established algorithm was used for diagnostic consensus concerning the two pathologists. All pathology slides were revised by a trained pathologist at ICO. The two pathologists reviewed the cases with discordant diagnosis and settled the diagnosis for a final evaluation. 

### 2.3. Samples Further Processing and HPV Genotyping 

FFPE-embedded samples were processed as previously described [27]. Total DNA was isolated using Maxwell^®^ RSC DNA FFPE Kit (Promega, Madison, WI, USA), and HPV positivity and genotyping was tested before viral load determination using Anyplex™ II HPV28 Detection (Seegene Inc., Seoul, Republic of Korea) assay and following manufacturers’ instructions. Anyplex™ II HPV28 Detection (Seegene Inc.) assay detects 28 HPV types including high-risk types and low-risk types (6, 11, 16, 18, 26, 31, 33, 35, 39, 40, 42, 43, 44, 45, 51, 52, 53, 54, 56, 58, 59, 61, 66, 68, 69, 70, 73, 82). All HPV11 or HPV6 positive samples were included in the viral load analysis.

### 2.4. HPV-DNA Detection, Genotyping, and Viral Load Determination 

Custom TaqMan assays (Applied Biosystems) targeting HPV E6 gene of either HPV-6 or HPV-11, and previously designed by Forslund 2005 and Pan 2004 [24,25], respectively, were used to determine HPV copies. RNaseP TaqMan Copy number reference assay (applied biosystems) was used to determine the number of cells, assuming 2 copies of the *RPPH1* gene per cell.

HPV load was performed using the DNA leftover obtained for HPV testing and genotyping. 

In total, 20 µL qPCR reactions containing 10 µL EagleTaq Master mix, 1 µL TaqMan assay, and 9 µL sample DNA were performed. DNA from 10^4^ HaCaT cells was added to each qPCR reaction as background DNA. All qPCR reactions were carried out in triplicate in a CFX96 (Bio-Rad) as follows: 95 °C for 10 min, 45 cycles at 95 °C for 15 s, at 60 °C for 60 s, and at 72 °C for 1 s. 

The number of copies of each target gene was obtained by linear interpolation from Cts and copy numbers obtained in regression standard curves. DNA from plasmids containing HPV-6 and HPV-11 genomes (ATCC-45150 and -45151, respectively) was used to obtain linear regression standard curves for absolute quantification of HPV-6 and HPV-11 copies. Viral load was described as HPV E6 copies per cell.

### 2.5. Statistical Analysis

Descriptive statistics were calculated for each of the variables studied. Fisher’s exact tests were used for categorical variables to analyse statistically significant differences between DSNEP and FSNEP cases, and (Student’s) *t*-test for continuous variables. Follow-up and time to recurrence were estimated. The statistical significance was established at *p* 0.05, adjusted by Bonferroni correction in case of characteristics with multiple choice options. The Cohen’s D index was calculated for continuous variables and Cramér’s V index for nominal variables to measure the effect size. All the analyses were performed with STATA 16.0 software.

## 3. Results

**Figure 2:**
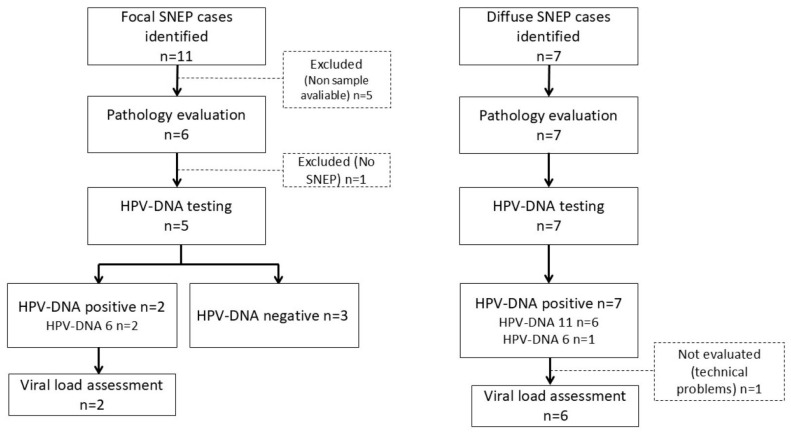
Flow chart of cases included in the study.

Eighteen SNEP patients diagnosed between 2013 and 2019 were selected and reviewed from the files of the pathology department in our centre. A total of 12 cases, from which FFPE samples were available, were included in the histopathological analysis and, finally, 12 were processed and tested (Figure 2). 

The characteristics of the patients are presented in Table 1. Most patients were males (83.3%) and ever-smokers (66.7%) with a mean age of 52.1 years and a median age of 48.5 years (range 30–92). None of them had a history of known HPV infection or immunosuppression. The majority of SNEP patients complained of nasal blockage at the diagnosis (75%) but others had rhinorrea (16.7%), epistaxis (16.7%), otitis media with effusion (8.3%), or no symptoms at all (8.3%). The most common localization of SNEP was in the septum (58.3%), followed by the inferior turbinate head (41.7%), the cupula of the nasal fossae (33.3%), and the lateral wall (33.3%). Less frequently, SNEP affected the floor of the nasal fossae and the middle turbinate head (two cases in each localization). Preoperative imaging was performed in 10 patients (83.3%) by CT scan. MRI was not used in any case. No imaging was performed in two patients (one FSNEP and one DSNEP) due to the small size of the lesions and its anterior location in the nasal fossae. CT scans showed swelling of the sinonasal mucosa (80%), sinus occupation (50%), and polipoid formation in the nasal fossae (40%). Diffuse nasal presentation were diagnosed in seven cases (58.3%). Seven patients (58.3%) had recurrence with a median time of recurrence of 5 months (range 1–67). The median time of follow-up was 46 months (range 4–100). No malignant transformation was detected during the follow-up. HPV-DNA prevalence was 75% (50% positive for HPV11, 25% positive for HPV6). Although histopathological evaluation confirmed that all 12 samples were exophytic nasal papillomas, minority foci of inverted papilloma were found in three cases.

Viral load of HPV6 positive cases ranged from 80.2 to 1169.9 copies/cell with a median of 83.9 copies/cell (mean of 444.6 copies/cell with a standard deviation of 627.9), and in HPV11 positive cases, ranged from 1058.7 to 3087.5 copies/cell with a median of 1673.7 copies/cell (mean of 1904.2 copies/cell and a standard deviation of 780.5). Viral loads were not evaluated due to technical problems in one HPV11 positive case (Table 2).

Statistically significant differences between patients affected by DSNEP and those affected by FSNEP were found for age (42.7 years vs. 65.2 years, respectively, *p* = 0.019). A strong association between age and focal/diffuse variables was observed (Cohen’s D index value of 1.61). A higher proportion of males and ever-smokers in the DSNEP group was noticed; however, these were not statistically significant (100% vs. 60%, *p* = 0.152, and 85.7% vs. 40%, *p* = 0.152, respectively). No statistically significant differences were found between focal and diffuse presentation regarding clinical symptoms. The nasal septum was more affected in DSNEP (85.7% vs. 20%, *p* = 0.029), while FSNEP appeared more frequently in the lateral wall (40% vs. 28.6%, *p* = 0.692) and the cupula (40% vs. 28.6%, *p* = 0.692) of the nasal cavity, although these differences were not statistically significant. In FSNEP patients, CT-scan imaging showed swelling of the sinonasal mucosa in three cases (75%), an occupation of the maxillary or ethmoidal sinuses in two cases (50%), and polipoid formation in the nasal fossae in one case (25%); while in DSNEP, the CT scan revealed mucosal swelling in five cases (83.3%), sinus occupation in three cases (50%), and polipoid formation in three cases (50%). No statistically significant differences were detected. A higher incidence of recurrences in DSNEP was found and it was statistically significant (85.7% vs. 20%, *p* = 0.045) compared with the focal group. The median time of recurrence were 67 months for the focal single case and 4 months (range 1–36) for the diffuse group (*p* = 0.134). No statistically significant differences were found in the follow-up (median of 43 months vs. 49 months, *p* = 0.745).

There were statistically significant differences between diffuse and focal SNEP regarding HPV detection (100% vs. 40%, respectively, *p* = 0.045) and specifically in HPV11 detection (85.7% vs. 0%, respectively, *p* = 0.008). The strength of association between diffuse presentation and recurrence, HPV positivity, and HPV11 were analysed, obtaining Cramér V index values of 0.66, 0.68, and 0.85, respectively. In all three cases, a strong association was observed. Among HPV6-positive SNEPs, the median viral load was higher in FSNEP than in DSNEP (626.8 vs. 80.2 virus/cell) (Table 2), while all HPV11-positive SNEPs were diffuse. A higher proportion of exophytic and inverted mixed papillomas were found in the focal group (40% vs. 14.3%, *p* = 0.364) although the difference was not statistically significant.

## 4. Discussion

SNEP is a rare clinical entity, and its diffuse behaviour is poorly described in the literature [4,8,10,28,29]. Herein, we identified, included, and collected follow-up data of all DSNEP and FSNEP cases consecutively diagnosed and treated in our centre during the study period and assessed the differences between both groups. To our knowledge, no other studies have compared such sociodemographic and clinical variables between DSNEP and FSNEPs. Moreover, our study is the first to analyse HPV viral load in the SNEP setting.

Our results regarding age of SNEP presentation is similar to other published articles [2,3]. Diffuse presentation was more commonly found in younger SNEP patients in our series. Glâtre et al. [8] described the ages of SNEP patients at an individual level but did not analyse age differences between FSNEP and DSNEP. 

Only one patient with FSNEP had a single recurrence (20%) while almost all DSNEP patients (85.7%) had recurrences. In the previous literature, the recurrence rate of focal SNEPs was estimated at around 22% [3,6], and Glâtre reported an 83.3% of recurrence in DNSEP and no recurrences in FSNEP [8]. Clinical symptoms and findings in CT scans in SNEPs were similar as those previously described [8], with no differences between focal and diffuse patterns. No histological differences between FSNEPs and DSNEPs were observed in our study. 

The location of SNEPs in our study was in accordance with others’ results [3,8,30]. In our series, DSNEPs were more frequently observed in the septum while FSNEPs affected mostly the lateral wall and the cupula of the nasal fossae. To the best of our knowledge, there is no information about nasal involvement in the literature. 

Observed HPV prevalences (40% in FSNEP and 100% in DSNEP) are consistent with previous studies [4,23,24], although there is a wide variability present in the literature [3,5,8,25]. DSNEP seemed to be related to an increased HPV prevalence, mostly with HPV11 genotype. Glatre et al. [8] reported similar data, with 100% of HPV11 positivity in DSNEP and 50% of coinfection with HPV6. Previous reports showed that in other benign lesions associated to HPV, such as recurrent respiratory papillomatosis (RRP), HPV11 presence is related to worse prognosis [31,32], while others report that the HPV genotype does not influence the outcome [33,34,35]. No cases of coinfection were found in our series.

We observed a tendency of a higher viral load in FSNEP within the HPV6-positive subgroup, but we are comparing only three samples, and thus, it is not possible to conclude anything in this subject. Despite this, there are no previous studies in the literature on viral load results in SNEPs and this can be a precedent for further studies. In the RRP setting, some reports state that viral load decreased with each treatment until remission was achieved [36], while other studies suggested that viral load is not related to the severity of RRP [37,38]. We could hypothesize that the viral load may be an indicator of virus activity in SNEP and of a higher risk for presenting a more aggressive or diffuse lesion, but larger studies are needed to assess that. We did not analyse the viral load during all recurrences to check its evolution during the follow-up, as has been performed in RRP and anogenital wart studies [36,39]. 

Our study has several limitations, with the small sample size due to the low prevalence of this pathology being the most important one. However, the numbers are similar to previous articles [3,4,8,25] and reflect the reality of this pathology in our real-world setting. Therefore, the results observed could set the basis for other further studies including larger populations. Mixed SNEPs were not excluded because, in all cases, the exophytic component presented a clear predominance over the inverted one, and it is common to observe inverted areas in SNEPs. The outcomes of our analysis must be considered with caution because of the small sample size and, consequently, its limited statistical power. Moreover, the number of recurrences may be determined because of the higher risk in interventions involving multiple lesions. P16 expression was not evaluated. However, for HPV6 or 11-positive and HPV-negative benign and premalignant lesions of the tonsil and larynx, p16 immunostaining is highly variable and is not thus recommended to predict HPV-presence. Our research can serve as a precedent for other studies to understand the physiopathology of DSNEP and to determine additional molecular factors that explain its behavior. Regarding DSNEP treatment, investigation into adjuvant drugs would be useful to reduce the number of surgeries needed and their side effects.

## 5. Conclusions

DSNEPs seem to be related to younger patients, the involvement of the nasal septum, the presence of HPV, mostly HPV11, and a higher risk of recurrence. Further research with larger numbers of cases is needed to unequivocally assess the risk factors associated with diffuse behaviour in the SNEP setting.

## Figures and Tables

**Table 1:** Demographic, clinical histological, and HPV characteristics of SNEP patients.

Characteristics	Cases (N = 12) n (%)	FSNEP (N = 5) n (%)	DSNEP (N = 7) n (%)	*p*-Value
**Gender**				
Male	10 (83.3)	3 (60.0)	7 (100.0)	0.152
Female	2 (16.7)	2 (40.0)	0	
**Age (years)**				
Mean	52.1	65.2	42.7	
Rank	30–92	51–92	30–62	**0.019**
Median	48.5	54	42	
**Smoking status**				
Ever	8 (66.7)	2 (40.0)	6 (85.7)	0.152
Never	4 (33.3)	3 (60.0)	1 (14.3)	
Clinical symptoms *				
Nasal blockage	9 (75)	4 (80)	5 (71.4)	0.636 ^(1)^
Rhinorrea	2 (16.7)	1 (20)	1 (14.3)	0.682 ^(1)^
Epistaxis	2 (16.7)	1 (20)	1 (14.3)	0.682 ^(1)^
Otitis media with effusion	1 (8.3)	1 (20)	0	0.417 ^(1)^
No symptoms	1 (8.3)	0 (20)	1 (14.3)	0.583 ^(1)^
Localization *				
Septum	7 (58.3)	1 (20.0)	6 (85.7)	0.045 ^(2)^
Inferior turbinate head	5 (41.7)	1 (20.0)	4 (57.1)	0.179 ^(2)^
Cupula/roof	4 (33.3)	2 (40.0)	2 (28.6)	0.576 ^(2)^
Lateral wall	4 (33.3)	2 (40.0)	2 (28.6)	0.576 ^(2)^
Floor	2 (16.7)	0	2 (28.6)	0.318 ^(2)^
Middle turbinate head	2 (16.7)	0	2 (28.6)	0.318 ^(2)^
Radiological characteristics *				
Swelling of sinonasal mucosa	8 (80)	3 (75)	5 (83.3)	0.576 ^(3)^
Sinus occupation	5 (50)	2 (50)	3 (50)	0.689 ^(3)^
Polipoid formation in the fossae	4 (40)	1 (25)	3 (50)	0.424 ^(3)^
No CT scan	2 (16.7)	1 (20)	1 (14.3)	0.682 ^(3)^
**Behaviour**				
Focal	5 (41.7)			
Diffuse	7 (58.3)			
**Recurrence**				0.045
Yes	7 (58.3)	1 (20%)	6 (85.7)	
No	5 (41.7)	4 (80%)	1 (14.3)	
**Time to recurrence (months)**				
Median	5	67	4	0.134
Rank	1–67	67	1–36	
**Follow-up (months)**				
Median	46	43	49	0.745
Rank	4–100	4–82	20–100	
**HPV status**				
Negative	3 (25.0)	3 (60.0)	0	**0.045**
Positive	9 (75.0)	2 (40.0)	7 (100.0)	
**HPV genotype**				
HPV11	6 (50.0)	0	6 (85.7)	**0.008 ^(4)^**
HPV6	3 (25.0)	2 (40.0)	1 (14.3)	0.364 ^(4)^
Negative	3 (25.0)	3 (60.0)	0	0.045 **^(4)^**
**Histology**				
Exophytic nasal papilloma	9 (75)	3 (60%)	6 (85.7)	0.364
Exophytic and inverted mixed papilloma	3 (25)	2 (40%)	1 (14.3)	

* Each case may fit in more than one item. In these cases, *p*-valor, to be considered for founding statistical differences, was adjusted with a Bonferroni correction: (1) *p* = 0.01; (2) *p* = 0.008; (3) *p* = 0.013; (4) *p* = 0.017. In bold, statistically significant differences. FSNEP: focal sinonasal exophytic papilloma, DSNEP: diffuse sinonasal exophytic papilloma.

**Table 2:** HPV-DNA genotyping and viral load results.

Patient	Behaviour	HPV Genotype	Viral Load			
			Copies/Cell	Mean	Sdt	Median
1	FSNEP	Neg	NA	-	-	-
2	FSNEP	Neg	NA			
3	FSNEP	Neg	NA			
4	FSNEP	HPV-6	83.9	444.6	627.9	83.9
5	FNSEP	HPV-6	1169.9			
6	DSNEP	HPV-6	80.2			
7	DSNEP	HPV-11	1058.7	1904.2	780.5	1673.7
8	DSNEP	HPV-11	1488.4			
9	DSNEP	HPV-11	1673.7			
10	DSNEP	HPV-11	2212.9			
11	DSNEP	HPV-11	3087.5			
12	DSNEP	HPV-11	NE	NE	NE	NE

NA: not applicable. NE: not evaluated due to technical problems. FSNEP: focal sinonasal exophytic papilloma, DSNEP: diffuse sinonasal exophytic papilloma.

## Data Availability

The original contributions presented in the study are included in the article/supplementary material; further inquiries can be directed to the corresponding authors.

## Supplementary Materials

The following supporting information can be downloaded at: https://www.mdpi.com/article/10.3390/jcm13226638/s1, Annex 1. Histological evaluation form.
