# Supplementary material for: Clinical, Histological, and HPV-Related Factors Associated to Diffuse Presentation of Exophytic Nasal Papillomas
Source: J Clin Med. 2024 Nov 5;13(22):6638. doi: 10.3390/jcm13226638 (PMC11594321; doi:10.3390/jcm13226638)
Supplement: Supplementary file 1 [file jcm-13-06638-s001.zip › jcm-3241855-supplementary.pdf]

Annex 1. HISTOLOGICAL EVALUATION FORM

Identification number ________ Date ______ Pathologist ________

1. HISTOLOGICAL DESCRIPTION OF THE LESION

1.1. Types of epithelia in the sinonasal exophytic papilloma (SNEP)

□ Squamous

□ Columnar

□ Presence of oncocytic cells

□ Hyper-parakeratosis

□ Presence of exophytic or papillary lesion adjacent to SNIP

□ Dysplasia □ Mild □ Moderate □ Severe/Carcinoma *in situ*

Others:

1.2. Intralesional polymorphonuclear infiltrate:

□ No

□ Yes □ Mild □ Moderate □ Severe

Inflammatory Perilesional infiltrate:

□ No

□ Yes □ Mild □ Moderate □ Severe

1.3. Subepithelial stromal tissue

□ Lax □ Dense

1.4. Other findings:

2. DEFINITIVE DIAGNOSIS OF THE LESION

□ Inverted

□ Oncocytic

□ Exophytic

□ Non-papillary lesion:

□ Dysplasia □ Mild □ Moderate □ Severe/Carcinoma *in situ*

3. Slides A&B

◻ Same ◻ Different

5. Control

◻ Tissue: ______________

6. Final evaluation

◻ Adequate for HPV analysis

◻ Repeat sandwich technique

◻ Doubtful/Uncertain

◻ Discard for HPV analysis

7. External quality control

◻ Pathologist ______________

8. Internal quality control

◻ Pathologist ______________

Comments

